# Polydatin Ameliorates Osteoporosis via Suppression of the Mitogen-Activated Protein Kinase Signaling Pathway

**DOI:** 10.3389/fcell.2021.730362

**Published:** 2021-09-29

**Authors:** Ze Lin, Yuan Xiong, Yiqiang Hu, Lang Chen, Adriana C. Panayi, Hang Xue, Wu Zhou, Chenchen Yan, Liangcong Hu, Xudong Xie, Yun Sun, Bobin Mi, Guohui Liu

**Affiliations:** ^1^Department of Orthopedics, Union Hospital, Tongji Medical College, Huazhong University of Science and Technology, Wuhan, China; ^2^Hubei Province Key Laboratory of Oral and Maxillofacial Development and Regeneration, Wuhan, China; ^3^Division of Plastic Surgery, Brigham and Women’s Hospital, Harvard Medical School, Boston, MA, United States

**Keywords:** MAPK, gene, osteoporosis, polydatin, KEGG pathway

## Abstract

**Purpose:** Polydatin (POL) is a natural active compound found in *Polygonum multiflorum* with reported anti-oxidant and antiviral effects. With the aging population there has been a stark increase in the prevalence of osteoporosis (OP), rendering it an imposing public health issue. The potential effect of POL as a therapy for OP remains unclear. Therefore, we sought to investigate the therapeutic effect of POL in OP and to elucidate the underlying signaling mechanisms in its regulatory process.

**Methods:** The POL-targeted genes interaction network was constructed using the Search Tool for Interacting Chemicals (STITCH) database, and the shared Kyoto Encyclopedia of Genes and Genomes (KEGG). Pathways involved in OP and POL-targeted genes were identified. Quantitative real-time PCR (qRT-PCR) and enzyme-linked immunosorbent assay (ELISA) were performed to evaluate the osteogenic genes and the phosphorylation level in pre-osteoblastic cells. In addition, ALP and alizarin red staining was used to test the effect of POL on extracellular matrix mineralization.

**Results:** Twenty-seven KEGG pathways shared between POL-related genes and OP were identified. *MAPK* signaling was identified as a potential key mechanism. *In vitro* results highlighted a definitive anti-OP effect of POL. The phosphorylation levels of *MAPK* signaling, including *p38*α, *ERK1/2*, and *JNK*, were significantly decreased in this regulatory process.

**Conclusion:** Our results suggest that POL has a promising therapeutic effect in OP. *MAPK* signaling may be the underlying mechanism in this effect, providing a novel sight in discovering new drugs for OP.

## Introduction

Osteoporosis (OP), a condition characterized by thin and brittle bones, compromises bone strength and predisposes bones to fractures, especially the bones in the hip, spine, and wrist (Cummings and Melton, 2002; Compston et al., 2019). The prevalence of OP is on the rise, owing to the aging population, with millions of people worldwide either already having OP or being at high risk due to low bone mass (Black and Rosen, 2016; Compston et al., 2019). Studies have suggested that approximately one in two women and up to one in four men aged 50 and older will suffer a bone fracture due to OP (Sambrook and Cooper, 2006; Rachner et al., 2011; Compston et al., 2019). Although immense strides have been made in drug development, the incidence of OP is growing exponentially (Rachner et al., 2011; Compston et al., 2019). Design and development of effective drugs that can delay the pathological progress of OP have the potential to revolutionize healthcare provision.

Owing to their low toxicity, natural active compounds of a plant origin are attracting attention (Zhu et al., 2018; Suroowan and Mahomoodally, 2019). Polydatin (POL, 3, 4, 5-trihydroxystibene-3-β-mono-D-glucoside), a stilbenoid compound obtained from the root of *Polygonum cuspidatum*, has a long history of use as a Chinese traditional medicine in a wide array of diseases (Chen et al., 2013; Jiang et al., 2013; Huang et al., 2015; Mele et al., 2018). Polydatin (POL) has been reported to enhance the anti-oxidant ability of bone marrow stromal cells (BMSCs) and to induce bone remodeling (Chen et al., 2019). A recent study reported that POL has anti-OP activity in ovariectomized mice (Shen et al., 2020). However, to the best of our knowledge, the mechanism of POL’s anti-OP activity remains elusive and requires further investigation.

Bioinformatic analyses have been widely applied in the elucidation of potential molecular mechanisms underlying diseases (Agarwal and Searls, 2009; Zampieri et al., 2017). In the current study, we employ a set of bioinformatic tools to identify the target genes and Kyoto Encyclopedia of Genes and Genomes (KEGG) pathways involved in POL’s mechanism of anti-OP activity. Our primary aim was to identify the potential molecular and cellular mechanism of POL in OP. We analyzed the shared KEGG pathways between POL-targeted genes and OP, and performed *in vitro* assays to validate our hypothesis.

## Materials and Methods

### Reagents

Polydatin was purchased from MedChemExpress LLC (NJ, United States), the quantitative real-time PCR (qRT-PCR) kit was purchased from Thermo Fisher Scientific Co. (Boston, MA, United States). The enzyme-linked immunosorbent assay (ELISA) kits were purchased from R&D SYSTEMS Co. (p-p38α and p-ERK1/2, Emeryville, CA, United States), and Shanghai Jianglai Ltd. (p-JNK, Shanghai, China).

### Culture of MC3T3-E1 Cells

Murine pre-osteoblasts (MC3T3-E1 cells) were kindly donated by the Shanghai University of Medicine & Health Sciences (Shanghai, China). The medium used for cell culture is α-MEMcontaining 10% FBS, and 1% penicillin and streptomycin. The cells were grown at 37°C with 5% CO_2_ at 95% humidity and were used for up to five passages. To induce a cellular OP model the MC3T3-E1 cells were treated with 100 μM dexamethasone (DXM) for 7 days.

### Quantitative Real-Time PCR Analysis

TRIzol was used for RNA extraction, according to the manufacturer’s protocol. cDNA was generated with a one-step Prime Script miRNA cDNA synthesis kit, and amplification of equivalent cDNA amounts was performed by SYBR Premix Ex TaqII. The qPCR analysis was performed by using a Thermal Cycler C-1000 Touch system. The reverse transcription-quantitative polymerase chain reaction messenger RNA quality of each gene was calculated using the 2^–ΔΔCt^ method and normalized to GAPDH. The primer sequences of the genes are displayed in Table 1.

**TABLE 1:** mRNA primer sequences.

**microRNA or gene names**	**Primer sequence (5′–3′)**
Mmu-Col-1a1-Forward	CTGACTGGAAGAGCGGAGAG
Mmu-Col-1a1-Reverse	CGGCTGAGTAGGGAACACAC
Mmu-ALP-Forward	TGACTACCACTCGGGTGAACC
Mmu-ALP-Reverse	TGATATGCGATGTCCTTGCAG
Mmu-OCN-Forward	TTCTGCTCACTCTGCTGACCC
Mmu-OCN-Reverse	CTGATAGCTCGTCACAAGCAGG
Mmu-Runx2-Forward	CGCCACCACTCACTACCACAC
Mmu-Runx2-Reverse	TGGATTTAATAGCGTGCTGCC
Mmu-GAPDH-Forward	TGAAGGGTGGAGCCAAAAG
Mmu-GAPDH-Reverse	AGTCTTCTGGGTGGCAGTGAT

### Enzyme-Linked Immunosorbent Assay

MC3T3-E1 were incubated serum-free medium for a 48-h period. The concentration of proteins was measured by ELISA. Before the ELISA assay, the number of cells in each culture well was counted to ensure that the cell numbers were same. The concentration of phospho-p38α, phospho-ERK1/2 and phospho-JNK were calculated based on the standard curve.

### ALP Staining

An ALP staining was performed by using the color-development kit based on the provided guidance to evaluate ALP staining results. Briefly, MC3T3-E1 cells were fixed in 10% formalin for 15 min after washing the cells twice with PBS. The BCIP/NBT liquid substrate was used to stain cells for 24 h. Absorbance was measured at 405 nm.

### Alizarin Red Staining

Cells were grown in six-well plates in a special osteogenic media (#HUXMA-90021, Cyagen, United States) for 21 days to promote osteogenesis. Briefly, cells were washed twice with PBS, followed by fixation in 10% formalin for 15 min. The cells were stained with 0.5% Alizarin-Red solution at room temperature for 15 minutes, then rinsed with distilled water for 5 min. A charge-coupled device microscope was used to analyzed red mineralized nodules. Absorbance was measured at 570 nm.

### Retrieval of Polydatin-Related Genes and Compounds

The Search Tool for Interacting Chemicals (STITCH) database^1^ was used to search for POL-related genes and compounds. STITCH is a database of known and predicted interactions between chemicals and proteins (Szklarczyk et al., 2016). POL-related genes and compounds were obtained using the following settings: the maximum number of interactions in each shell was 10, three shells were retrieved, and the intermediate confidence score was 0.4. The data were imported into Cytoscape 3.8.0 to construct a POL-related gene relationship network and to calculate the degree, betweenness, and closeness of each gene in the network. A weighted network was constructed according to the degree of genes in Cytoscape (Shannon et al., 2003).

### Enrichment Analysis of Genes and Kyoto Encyclopedia of Genes and Genomes Pathways

The database for Annotation, Visualization, and Integrated Discovery (DAVID) database was used to search POL-related KEGG pathway. The DAVID knowledge base contains millions of identifiers from thousands of species allowing agglomeration of a diverse array of functional and sequence annotation, greatly enriching the level of biological information available for each gene (Huang et al., 2009; Jiao et al., 2012).

### Shared Kyoto Encyclopedia of Genes and Genomes Pathways

The miRwalk2.0 database was used to search for KEGG pathway related to OP (Dweep and Gretz, 2015). POL targeted gene related KEGG pathways were also identified (*q* ≤ 0.05). The shared KEGG pathways were established with a Venn Diagram (Venny 2.1^2^).

### Identification of the Hub Genes

Gplot, an R package that visually combines expression data with functional analysis, was used to present the enrichment information of the top five KEGG pathways (Walter et al., 2015). The genes included in the top five KEGG pathways were considered hub genes. The specific information and chromosomal position of all genes in the network were presented using the circlize R package (Gu et al., 2014).

### Retrieval of the Kyoto Encyclopedia of Genes and Genomes Pathway

The top five shared KEGG pathways with the smallest *q*-values were selected and the KEGG pathways were established using the KEGG database.^3^

### Statistical Analysis

All analyses were conducted by GraphPad Prism 8.0; the presentation of data is mean ± SD. The data of two groups were compared with Student’s *t*-test, whereas one-way analysis of variance with Tukey’s textitpost-hoc test was used to compare groups of 3 or more. *P* < 0.05 was considered to be statistically significant. All experiments were repeated in triplicate.

## Results

### Polydatin-Related Genes and Interaction Network

**FIGURE 1:**
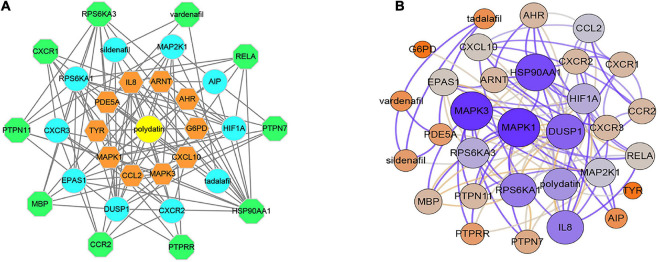
Interaction networks of polydatin-targeted genes. **(A)** Interaction network constructed by Cytoscape. **(B)** Weighted interaction network.

In total, 30 POL genes and compounds were obtained in STITCH using a limit of three shells. The interaction network was constructed in Cytoscape (Figure 1A). IL8, ARNT, AHR, G6PD, CXCL10, MAPK3, CCL2, MAPK1, TYR, and PDE5A were involved in the first shell. Sildenafil, MAP2K1, AIPm HIF1A, tadalafil, CXCR2, DUSP1, EPAS1, CXCR3, and RPS6KA1 were involved in the second shell. RPS6KA3, vardenafil, RELA, PTPN7, HSP90AA1, PTPRR, CCR2, MBP, PTPN11, and CXCR1 were involved in the third shell. A weighted network was constructed (Figure 1B). MAPK1 and MAPK3 had the highest weight.

DAVID database was used to obtain 69 polydatin-related KEGG pathways and 61 KEGG pathways with *q*-value < 0.05 were selected. And the miRwalk database was used to obtain 110 osteoporosis-related KEGG pathways.

### Enrichment Analysis of Genes and Kyoto Encyclopedia of Genes and Genomes Pathway

**FIGURE 2:**
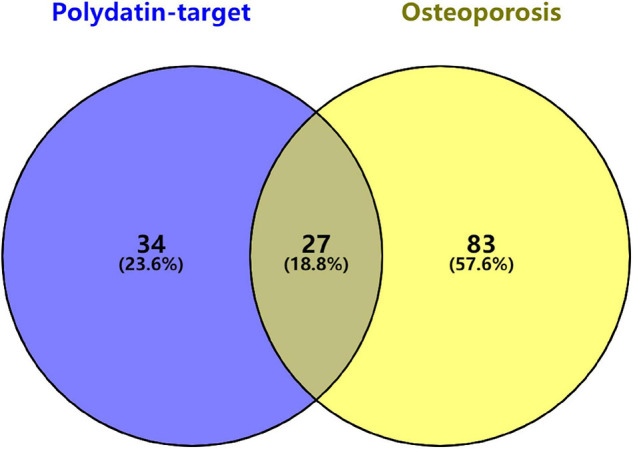
Identification of KEGG pathways shared between polydatin-targeted genes and osteoporosis. Sixty-one polydatin-targeted genes related KEGG pathways, and 110 osteoporosis related KEGG pathways were found; 27 (18.8%) shared KEGG pathways were identified.

**FIGURE 3:**
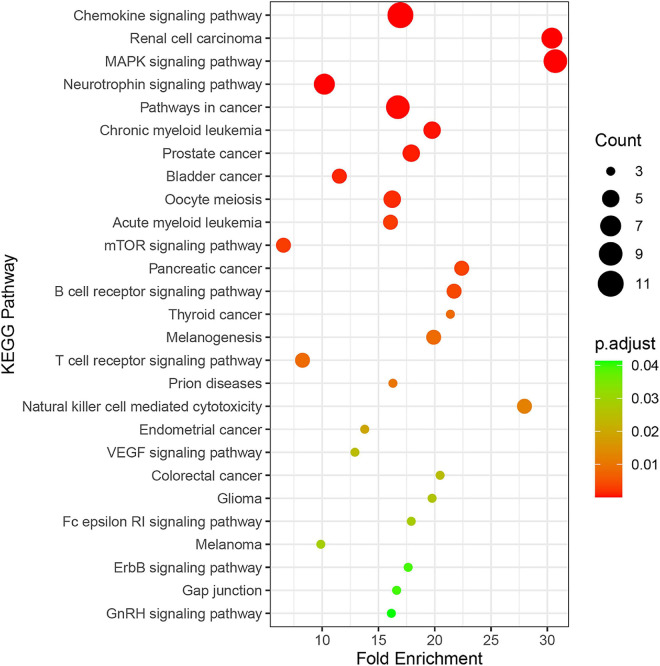
Enrichment information of KEGG pathways with *p*.adjust < 0.05. Top five KEGG pathways were the Chemokine signaling pathway (hsa04062), Renal cell carcinoma (hsa05211), the MAPK signaling pathway (hsa04010), the Neurotrophin signaling pathway (hsa04722), and Pathways in cancer (hsa052009).

**FIGURE 4:**
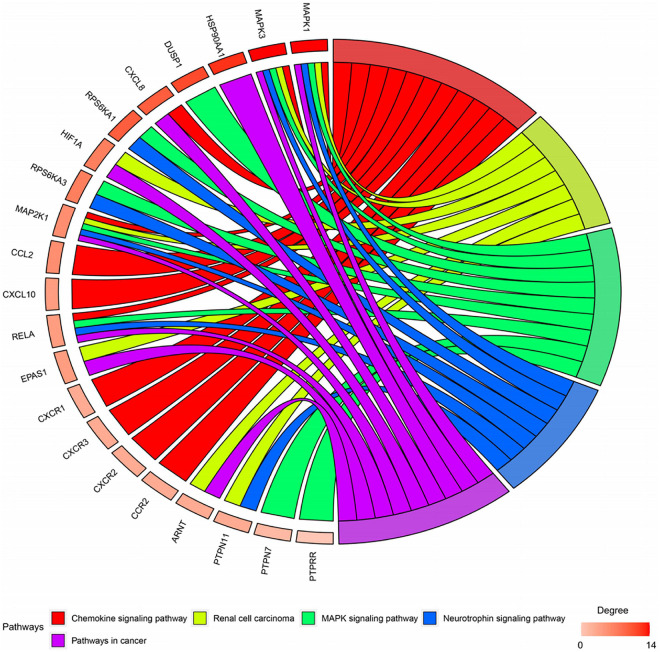
Gene enrichment analysis. MAP2K1, MAPK1, and MAPK3 were involved in all five pathways. The sub genes by degree were MAPK1, MAPK3, and MAP2K1.

**FIGURE 5:**
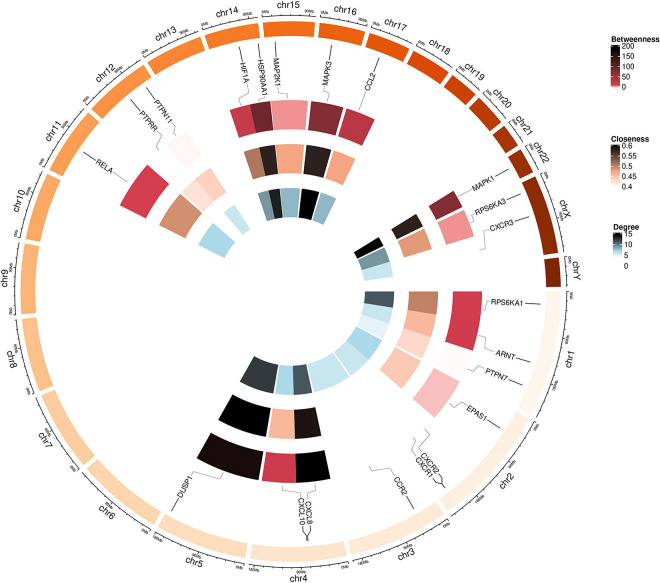
Circular visualization of the chromosomal positions and connectivity of polydatin-targeted genes. Gene names are shown in the outer circle. In the outer heatmap, deep color represents high betweenness, in the middle heatmap, deep color represents high closeness, and in the inner heatmap, deep color represents high degree. Lines extend from each gene point to its specific chromosomal locatio on the chromosomal circle.

Twenty-seven KEGG pathways shared between POL-related genes and OP were identified using a Venn Diagram (Figure 2). According to the above analysis, the top five KEGG pathways were Chemokine signaling pathway, Renal cell carcinoma, MAPK signaling pathway, Neurotrophin signaling pathway, and Pathways in cancer (Table 2). According to the information in the table, MAPK1, MAPK3, and MAP2K1 are found in all top five KEGG pathways. Therefore, these genes are regarded as hub genes. The enrichment information of the KEGG pathways with p.adjust < 0.05 is shown in Figure 3. The gene enrichment analysis results are shown in Figure 4. The specific information and the chromosomal position of all genes in the network are shown in Figure 5.

**TABLE 2:** Top five KEGG pathway and related genes.

**Term**	**KEGG pathway**	**Polydatin-targeted genes**	**q-value**
hsa04062	Chemokine signaling pathway	CXCL10, MAP2K1, CXCL8, CXCR1, CXCR3, CXCR2, MAPK1, CCL2, RELA, CCR2, MAPK3	9.52E-09
hsa05211	Renal cell carcinoma	MAP2K1, EPAS1, ARNT, MAPK1, PTPN11, HIF1A, MAPK3	1.90E-06
hsa04010	MAPK signaling pathway	RPS6KA3, MAP2K1, PTPRR, DUSP1, RPS6KA1, MAPK1, PTPN7, RELA, MAPK3	1.57E-05
hsa04722	Neurotrophin signaling pathway	RPS6KA3, MAP2K1, RPS6KA1, MAPK1, PTPN11, RELA, MAPK3	2.72E-05
hsa05200	Pathways in cancer	MAP2K1, HSP90AA1, CXCL8, EPAS1, ARNT, MAPK1, HIF1A, RELA, MAPK3	1.52E-04

### Retrieval of the Kyoto Encyclopedia of Genes and Genomes Pathway

**FIGURE 6:**
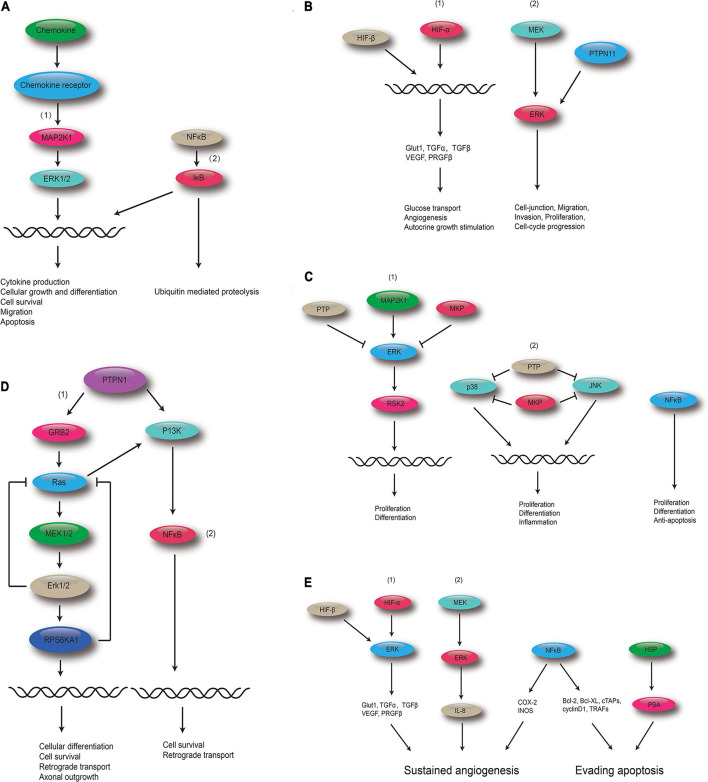
Polydatin-targeted genes related to the top five shared KEGG pathways. **(A)** Polydatin-targeted genes related to the Chemokine signaling pathway: (1) MAPK signaling pathway and (2) NF-kB. **(B)** Polydatin-targeted genes related to Renal cell carcinoma: (1) HIF-1 signaling pathway, and (2) MAPK signaling pathway. **(C)** Polydatin-targeted genes related to the MAPK signaling pathway:(1) Classical MAP kinase pathway, (2) JUK and p38 MAP kinase pathway, and (3) NFκB. **(D)** Polydatin-targeted genes related to the Neurotrophin signaling pathway: (1) MAPK signaling pathway, and (2) NFκB. **(E)** Polydatin-targeted genes related to Pathways in cancer: (1) HIF-1 signaling pathway, (2) MAKP signaling pathway, (3) NFκB, and (4) HSP.

The top five shared KEGG pathways with the smallest q-values are shown in Figure 6. These pathways are involved in proliferation, invasion, differentiation, inflammation, and cell survival. The MAPK signaling pathway is found in all the top five shared KEGG pathways.

### Polydatin Reverses Osteoporosis *in vitro*

**FIGURE 7:**
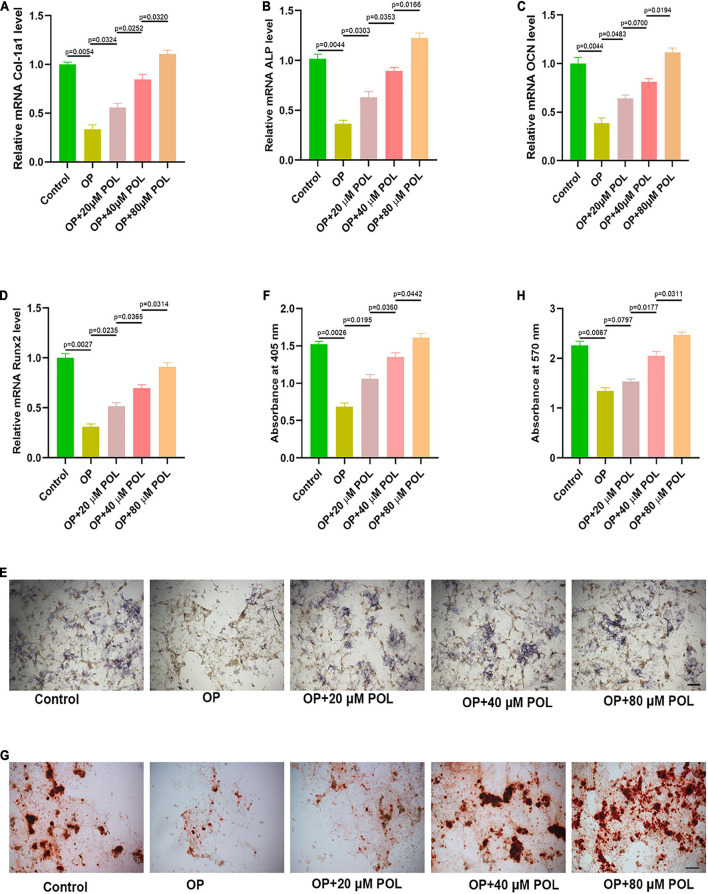
Polydatin (POL) reverses OP *in vitro*. **(A–D)** The expression of Col-1a1, ALP, OCN, and Runx2 in the different groups [Control(PBS); OP; OP + 20μM POL; OP + 40μM POL; OP + 80μM POL] was measured using a qRT-PCR analysis. The cellular OP model was performed using DXM; **(E,F)** ALP staining in MC3T3-E1 following different treatments; **(G,H)** Alizarin red- calcium staining in MC3T3-E1 following different treatments.

A cellular OP model was created using DXM. The MC3T3-E1 cells were treated with POL in different concentrations (20, 40, and 80 μM), the total RNA was extracted and the levels of osteogenic genes, including Col-1a1, ALP, OCN, and Runx2 were measured using qRT-PCR analysis. Our results showed that the DXM treatment could significantly decreased the bone turnover markers in MC3T3-E1 cells, and POL could partially reverse this effect in a dose-dependent manner (Figures 7A–D). Additionally, ALP staining was performed to visualize the extracellular matrix mineralization among the different groups. Similarly, POL could partially rescue the impaired mineralization induced by the DXM treatment (Figures 7E–H).

### MAPK Signaling Pathway Involved in the Regulation of POL

**FIGURE 8:**
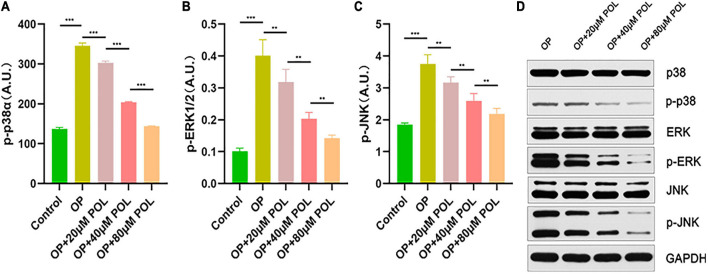
Involvement of the MAPK signaling pathway in the regulation of POL. The relative expression of **(A)** p-P38, **(B)** p-ERK, and **(C)** p-JNK in the different groups [Control (PBS); OP; OP + 20μM POL; OP + 40μM POL; OP + 80μM POL] by ELISA is shown (**P < 0.05, ***P < 0.001). **(D)** The cellular OP models were treated with different concentrations of POL. Western blotting results of p38, p-p38, ERK, p-ERK, JNK, and p-JNK protein levels in BMSCs.

As shown in Figure 8, in the POL-treated groups, the relative expression of p-JNK, p-P38, and p-ERK was decreased compared to the control group (PBS treatment) in a dose-dependent manner. Thus, it can be assumed that the *MAPK* signaling pathway is involved in the regulation of POL.

## Discussion

Polydatin, a stilbenoid compound obtained from the root of *P. cuspidatum*, is believed to possess anti-osteoporotic activity (Chen et al., 2016, 2019; Zhou et al., 2016). BMSCs have the ability of self-renewal and multidirectional differentiation. They can potentially differentiate into adipocytes, osteoblasts, and chondrocytes (Yim et al., 2014; Ruiz et al., 2016). Therefore, BMSCs play a key role in the treatment of OP. As shown in previous study, POL can protect from oxidative stress and promote BMSCs migration (Chen et al., 2019). POL has also been shown to possess notable anti-OP activity via regulation of OPG, RANKL, and β-catenin (Zhou et al., 2016). In addition, a study suggested that POL may promote BMSC migration via the ERK 1/2 signaling pathways (Chen et al., 2016). However, the precise mechanism of POL’s anti-OP activity has yet to be investigated.

In this study, we identified 27 KEGG pathway shared between POL-targeted genes and OP. The top five KEGG pathways with the smallest q-values were Chemokine signaling pathway, Renal cell carcinoma, MAPK signaling pathway, Neurotrophin signaling pathway, and Pathways in cancer. The hub genes of the five signaling pathway were MAPK1, MAPK3, and MAP2K1.

By mapping the KEGG pathways related to target genes, we found that POL exerts its biological effects through regulating Classical *MAP* kinase pathway, *JNK*, and *p38 MAP* kinase pathway. POL-targeted genes *ERK (MAPK1, MAPK3), MEK1 (MAP2K1)* were involved in the above pathways. And the identified POL-targeted genes are associated with proliferation, differentiation, inflammation, cellular growth and differentiation, and cytokine production.

As is known, there are three major subfamilies of MAPK: the extracellular-signal-regulated kinases (ERK MAPK, Ras/Raf1/MEK/ERK), the c-Jun N-terminal or stress-activated protein kinases (JNK, SAPK), and p38 (Fang and Richardson, 2005; Cargnello and Roux, 2011). JNK and p38 have similar functions and are related to inflammation, apoptosis, and growth (Wagner and Nebreda, 2009). ERK is responsible for basic cell processes, including cell proliferation and differentiation (Guo et al., 2020). Several studies have suggested that the ERK-MAPK pathway can positively regulate bone development (Radio et al., 2006; Ge et al., 2007; Shim et al., 2013). At the same time, studies have shown that the p38 MAPK pathway is essential for bone production and bone homeostasis (Greenblatt et al., 2010; Weske et al., 2018). In addition, osteoclast formation and survival can be inhibited through the attenuation of JNK/c-jun and NFκB signaling (Abe et al., 2003; Krum et al., 2010).

Protein phosphorylation (PP) is a common regulatory mode in organism and plays an important role in the process of cell signal transduction (Kummer and Ban, 2021). It was widely demonstrated that PP is the most basic, universal and important mechanism for regulating and controlling protein activity and function (Jiang et al., 2021). To validate our bioinformatic results, the phosphorylation level of MAPK signaling pathway was detected. Our results indicated that POL reduced the phosphorylation levels of ERK1/2, p38α and JNK in MC3T3-E1, suggesting *MAPK* signaling pathway involved in the regulation of POL, which is high incidence with the bioinformatic results. In the current study, we proved that POL induces osteoblastic differentiation via suppressing the *MAPK* signaling pathway, and further signaling pathways involved in the protective functions of POL on OP will be verified in future studies.

Like other bioinformatic analysis, some limitations could be found in the study. On the one hand, the effect of activation or suppression of *MAPK* signaling on osteoblasitc differentiation was not explored in the present research. On the other hand, animal osteoporotic model was not constructed and the beneficial effect of POL on OP was nor demonstrated *in vivo*.

However, it is worth noting, that previous researches has identified POL as a potential activator of the Sirtuin family, which is involved in specific biological functions, including regulation of transcription, cell cycle, cell differentiation, apoptosis, anti-oxidation, and genomic stabilization (Chen and Lan, 2017; Sun et al., 2021). Sirtuins, which is highly conserved NAD+ dependent deacetylases, exist in most organisms and play a key role in promoting the health and survival (Sinclair and Guarente, 2006; Haigis and Sinclair, 2010). According to previous studies, sirtuins can regulate the lifespan of lower organisms and age-related diseases in mammals (Imai and Guarente, 2014). A study has shown that sirtuin might play an important role in the treatment of mitochondrial dysfunction, aging, and metabolic diseases (Westphal et al., 2007). As an activator of sirtuin, resveratrol can reduce oxidative stress and inflammation by acting on Akt and MAPK signaling pathways (Shin et al., 2012). A related study has reported that sirituin affects the MAPK pathway by regulating the phosphorylation of p38, JNK, and ERK (Becatti et al., 2012). Therefore, this evidence taken together with our results, allows for speculation that polydatin might alleviates osteoporosis by acting on the Sirtuin family and regulating biological processes and MAPK signaling. This highlights a potential path for subsequent research.

## Conclusion

Our study determined that POL exhibited protective effects in OP, as evidenced by a suppression of *MAPK* signaling *in vitro*. This study identifies a promising potential candidate for the treatment of OP.

## Data Availability Statement

The original contributions presented in the study are included in the article/supplementary material, further inquiries can be directed to the corresponding authors.

## Author Contributions

GL conceived and designed the study. BM and YS supervised the study. ZL, YX, and YH performed the bioinformatics analysis and experiments and wrote the manuscript. LC, WZ, and HX analyzed the data. LH and AP provided advice and technical assistance. CY and XX revised the figures and tables. All authors approved the final manuscript.

## Conflict of Interest

The authors declare that the research was conducted in the absence of any commercial or financial relationships that could be construed as a potential conflict of interest.

## Publisher’s Note

All claims expressed in this article are solely those of the authors and do not necessarily represent those of their affiliated organizations, or those of the publisher, the editors and the reviewers. Any product that may be evaluated in this article, or claim that may be made by its manufacturer, is not guaranteed or endorsed by the publisher.

## Footnotes

1
http://stitch.embl.de/

2
http://bioinfogp.cnb.csic.es/tools/venny/index.html

3
https://www.kegg.jp/

## Abbreviations

OP: osteoporosis
POL: polydatin
MAPK: mitogen-activated protein kinase
STITCH: Search Tool for Interacting Chemicals
KEGG: Kyoto Encyclopedia of Genes and Genomes
ELISA: enzyme-linked immunosorbent assay.
